# Functions and clinical significance of circular RNAs in glioma

**DOI:** 10.1186/s12943-019-1121-0

**Published:** 2020-02-15

**Authors:** Jikui Sun, Banban Li, Chang Shu, Quanfeng Ma, Jinhuan Wang

**Affiliations:** 1 School of Medicine, Nankai University, 94 Weijin Road, Nankai District, Tianjin, 300071 People’s Republic of China; 2 Tianjin Cerebral Vascular and Neural Degenerative Disease Key Laboratory, Tianjin Neurosurgery Institute, Department of Neurosurgery, Tianjin Huan Hu Hospital, Tianjin, 300350 People’s Republic of China; 3 Qilu Hospital, Shandong University, 107 Cultural West Road, Jinan, 250012 People’s Republic of China; 4 Department of Hematology, Taian Central Hospital, 29 Longtan Road, Taian, 271000 People’s Republic of China

**Keywords:** Circular RNAs, Glioma, Biomarker, Signaling pathways, Database

## Abstract

CircRNAs are a class of single-stranded RNA molecules with a covalently closed loop structure and have been characterized by high stability, abundance, conservation, and display tissue/developmental stage-specific expression, furthermore, based on the abundance in distinct body fluids or exosomes, circRNAs present novel biomarkers and targets for the diagnosis and prognosis of cancers. Recently, the regulatory mechanisms of biogenesis and molecular functions, including miRNAs and RBPs sponge, translation as well as transcriptional and splicing regulation, have been gradually uncovered, although various aspects remained to be elucidated in combination with deep-sequence and bioinformatics. Accumulating studies have indicated that circRNAs are more enriched in neuronal tissues partly due to the abundance of specific genes promoting circularization, suggesting dysregulation of circRNAs is closely related to diseases of the nervous system, including glioma. In this review, we elaborate on the biogenesis, functions, databases as well as novel advances especially involved in the molecular pathways, highlight its great value as diagnostic or therapeutic targets in glioma.

## Background

In the last several decades, scientific research for non-coding RNAs provided new insights for exploring comprehensive molecular mechanisms in glioma. Numerous studies demonstrated that non-coding RNAs play crucial roles in biological processes that regulated glioma initiation and progression [1]. Moreover, circular RNAs (circRNAs) have become new research hotspots following miRNAs, lncRNAs in recent years. CircRNAs were first discovered in plant Viroids and Sendai virus via electron microscopy as early as 1976 and thereafter in eukaryotic cells in 1979 [2–4]. Until 1991, endogenous circRNAs were first identified in the transcripts of the tumor suppressor gene DCC in humans [5]. Unlike the linear RNAs, circRNAs are covalently single-stranded closed circular transcripts lacking 5’caps and 3’tails, and they are often considered aberrant byproducts or ‘splicing noise’ with low abundance and little functional potential [6, 7]. However, with the rapid development of high-throughput sequencing (RNA-seq) and bioinformatics, as well as combined complication of various algorithms for circRNA detection and quantification with non-poly(A) RNAs, a large number of circRNAs from pre-mRNA back-splicing have been identified and annotated not only in normal tissues especially but also in distinct cancers. Xu et al. identified at least 1000circRNAs in each tissue from six types including liver, heart, lung, stomach, colon, kidney [8]. Agnieszka Rybak-Wolf uncovered abundant and specific expression of circRNAs in the brain through analyzing ribosomal-depleted RNA from 29 different stages of neural and tissues and also showed the high stability and incompatibility as compared to relevant linear mRNAs [9], suggesting the roles in the course of neural differentiation and neurological diseases. Josh N. Vo utilized an exome capture RNA sequencing protocol to detect and characterize circRNAs across greater than 2000 cancer sample and compiled a cancer circRNA landscape including brain cancer, lung cancer, thyroid cancer, breast cancer, bladder cancer [10], indicating that circRNAs are involved in the pathogenesis of a variety of cancers. Recent studies experimentally confirmed that circRNAs played significant roles in tumor growth, metastasis, EMT transformation, and therapy resistance [11].

CircRNAs are newly discovered to define as a type of non-coding RNA and highly conserved across multiple species, exhibiting tissue-specific, and development stage-dependent patterns [12–14]. However, in addition to potential function as “miRNA sponge”, transcriptional regulators, protein binding, strong evidence have confirmed quite a number of circRNAs could translate protein, whereas these encoded peptides act as a novel resource bank for drug targets [15, 16]. Multiple recent studies containing above mentioned verified that the circRNAs are more enriched in neuronal tissues compare with other issues. Brain-specific genes may obtain more sequence features that promote RNA circularization, and widespread regulations by cis-elements and trans-factors might result in a higher abundance of circrRNAs in the brain [17–19]. However, the abundance of circRNAs in glioma specimens is lower than normal samples, prompting us to consider the roles and potential clinical application for glioma progression [20]. A better understanding of the function and mechanism of circRNAs in glioma tumorigenesis may contribute to the development of novel detection methods and effective therapeutic measures. In this review, we survey current progress regarding the regulation of circRNA biogenesis and function and highlight the potential clinical implications of human circRNAs on glioma.

### Biogenesis and regulation of circRNAs

**Fig. 1:**
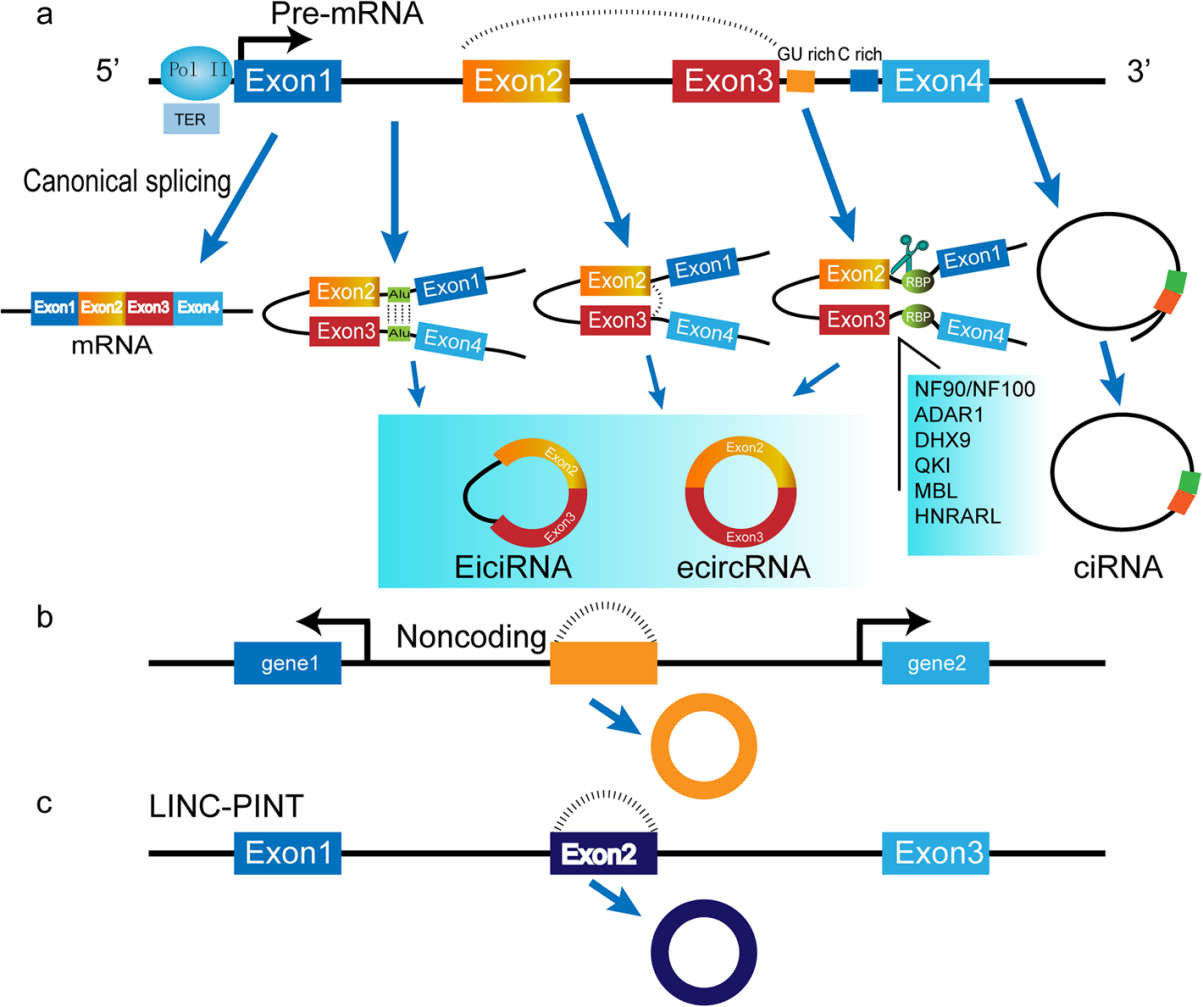
The representative summarized models of circRNA formation. **a** Three typical circularization forms. Intron pairing-driven circularization is mediated by cis-acting regulatory elements that include reverse complementary sequences (Alu repeats), contributing direct base pairing of flanking introns. Lariat driven circularization, lariats formation of pair intron make exon skipping through back splicing and lead to the formation of ecircRNA or EIciRNA. Trans-acting factors, such as RNA-binding proteins (RBPs) or several splicing factors that bind to specific sequence motifs of flanking introns, promote circRNA biogenesis. GU rich and C rich elements contribute to generating intronic RNA following the canonical splicing. **b** Intergenic circRNAs are generated from the distant regions between genes. **c** The formation of circular LINC-PINT exon

CircRNAs are highly stable RNAs that are resistant to exonucleases (RNase R) and are mainly generated by pre-mRNA back-splicing, which connects a downstream splice donor site (5’splice site) to an upstream acceptor splice site (3’splice site) [21–23]. More than of 80% circRNAs originate from exons of protein-coding genes which prominently are located in the cytoplasm, whereas single gene loci can generate multiple exon circularization patterns [24, 25]. According to biogenesis from different genomic regions, circRNAs could be classified into five types. (a) exonic circRNA (ecircRNNAs) containing one or usually multiple exons, usually two or three exons deriving from alternative splicing [24, 26]. Three models of ecircRNAs were presented, including lariat-driven circularization, intro-pairing-driving circularization and RNA binding protein (RBP) [27] (Fig. 1a). b. Intronic RNAs (ciRNAs) depending mainly on a 7 nt GU-rich element adjacent to the 5′ splice site and an 11 nt C-rich element adjacent to the branch point site, which is sensitive to RNA debranching enzymes [28] (Fig. 1a).c. exon-intron circRNAs (EIcircRNA) [29] (Fig. 1a).d. intergenic circRNAs [30] (Fig. 1b). In addition to these, there are reports of circRNAs generated from long ncRNAs that contain short open reading frames (sORFs), such as LINC-PINT, revealing the richness and complexity of RNA sources [31] (Fig. 1c).

RNA pol II can affect pre-mRNA processing via changing the transcription elongation rate (TER) (Fig. 1a), which controls the outcome of splicing events as an efficient modulator of alternative splicing. Zhang [32] studied that Fast Pol II Elongation Rate is positively associated with back-splicing circularization in agreement with reports from Fong [33], in addition, the mutation reducing TER in the fly produces a significantly lower number of circRNAs, even when normalized to the linear forms of the same gene, suggesting that circularization of exons is carried out by the canonical splicing machinery. Although resistance to exonucleolytic degradation allows circRNAs to accumulate to relatively high levels in cells, the efficiency of back-splicing circularization was predicted to be lower in general. The results from 4sUDRB-seq and qRT-PCR analyzing chromatin-bound (nascent) RNA strongly suggests that circRNAs are generated co-transcriptionally in the heads of flys and mammals, and pre-mRNA splicing can compete with circularization of exons [34]. Using RNAi screening, the study found that depletion of many core spliceosomal components, including the SF3b and SF3a complexes, resulted in specific increases in the steady-state levels of circRNAs from endogenous Drosophila genes, also indicating competition between them [35], however, from the reports of zhang, a few circRNAs could be produced co-transcriptionally within short 4sU labeling periods (10 and 15 min) after DRB removal, and the majority of circRNAs could be generated after the transcription of their parent genes has completed, suggesting that back-splicing primarily occurs post-transcriptionally. Based on controversial research, the special relationship between the splicing machinery and regular splicing activities, as well as structural elements affecting alternative splicing, remained to further deciphered. Enrichment of complementary Alu sequences and reverse-complementary repeats (PCR) in the long flanking introns all were able to promote the exon circularization [27, 36, 37]. Additionally, IRAlus pairing and other nonrepetitive complementary sequences in flanking Introns can promote circRNA formation. However, this type of AU repeats is not sufficient, a competition of RNA pairing within individual introns or across flanking introns preferentially facilitate exon circularization. The different distributions of ALUs or complementary sequences across species determine the distinction of the alternative splicing, also indicting that the machinery for circRNA formation is evolutionarily dynamic and complex in posttranscriptional regulation [26]. The influence of the length of the intron region has also been reported, for instance, mutational analyses of minigene demonstrated complementary short regions in the flanking introns induce efficient circularization which is inconsistent with the report from Ashwal-Fluss [26, 34, 38]. Besides, the structure of RCR, protein-mediated intron interactions may intimately be associated with the efficiency of circRNA, implying the complex of spliceosomal machinery regulation.

RNA-binding protein (RBPs), acting as trans-acting activators or inhibitors, has experimentally proved that they have significant roles in the regulation of circRNA production. Li et al. used genome-wide siRNA screening and an efficient circRNA expression reporter to identify over 100 double-stranded RNA-binding proteins for circRNA formation. The immune factors NF90/NF110 promoted circRNA production by binding to IRAlus and stabilizing intronic RNA pairs in the nucleus [39]. The RNA editing enzyme adenosine deaminase 1(ADAR1) inhibited circRNA production by directly weakening inverted ALU repeats through A-to-I editing of RNA pairing flanking circularized exons. Knockdown of ADAR1 stabilized intron base pairing interactions, up-regulated circRNA expression in neural tissue of flies and mammals [9, 26, 37]. Another example of Alu elements targets similar to ADAR1, DHX9, an abundant nuclear RNA helicase, explicitly bound to inverted-repeat Alu elements, and loss of DHX9 led to an increase in the number of circular RNAs. Besides, the co-depletion of ADAR and DHX9 augmented the double-stranded RNA accumulation defects, resulting in increased circRNA production in general, revealing a functional link between these two enzymes due to their relation to ALUs [40] (Fg.1a). Although these dsRBPs have been identified as effective regulators of circRNA formation in a genome-wide screen, the detail regulatory mechanisms need to be clarified.

Several splicing factors can also participate in circRNA formation regulation via indirectly or directly binding to specific RNA motifs. For example, Quaking (QKI), was proposed to bind to sites flanking introns and brought the circularized exons closer together, leading to circRNA upregulation during EMT. Insertion of QKI binding sites into linear RNA can induce exon circularization, revealing that circRNAs abundance is dependent on intronic QKI binding motifs [41]. YY1/p65/p300 complex could increase QKI expression through super-enhancer binding, suggesting an indirect regulatory role of the YY1 complex [42]. The splicing factor muscleblind (MBL/MBNL1) can promote the second exon circularization of its pre-mRNA through binding to multiple flanking intronic sequences. Further, downregulation of MBL in fly neural tissue resulted in a substantial decrease in circMbl production. In addition to the MBL binding sites that exist in the flanking introns, the mbl exon 2 itself is significantly abundant in putative MBL binding sites, suggesting MBL may bind directly to its exon and thereby regulated its circularization [34]. FUS was identified to regulate circRNAs biogenesis by binding the introns flanking the back-splicing junctions in murine embryonic stem cell-derived motor neurons [43]. Multiple hnRNP (heterogeneous nuclear ribonucleoprotein) and SR (serine–arginine) proteins regulated Laccase circular RNA levels that are not controlled by MBL in a combinatorial manner [44]. The heterogeneous nuclear ribonucleoprotein L (HNRNPL) also regulated circRNAs formation via back splicing by genome-wide CRISPR screen in human prostate cancer [45] (Fig. 1a). Altogether, it seemed that the complicated regulation of circRNA via different cis-elements and trans-elements is, to a large extent, dependent on biological settings and specification for back-splicing from host genes of various species. However, the specific mechanism involved biogenesis of circRNAs is elusive, and how related splicing factors regulate circRNAs formation needs to further investigate.

### Functions of circular RNAs

Multiple circRNAs have been verified to be expressed more abundantly independent of their linear counterparts and have a cell type and stage-specific manner [13, 46, 47], recently, increasing lines of evidence symbolized the complexity and importance for regulation of distinct biological processes.

### CircRNAs act as miRNA sponges or competing endogenous RNA

**Fig. 2:**
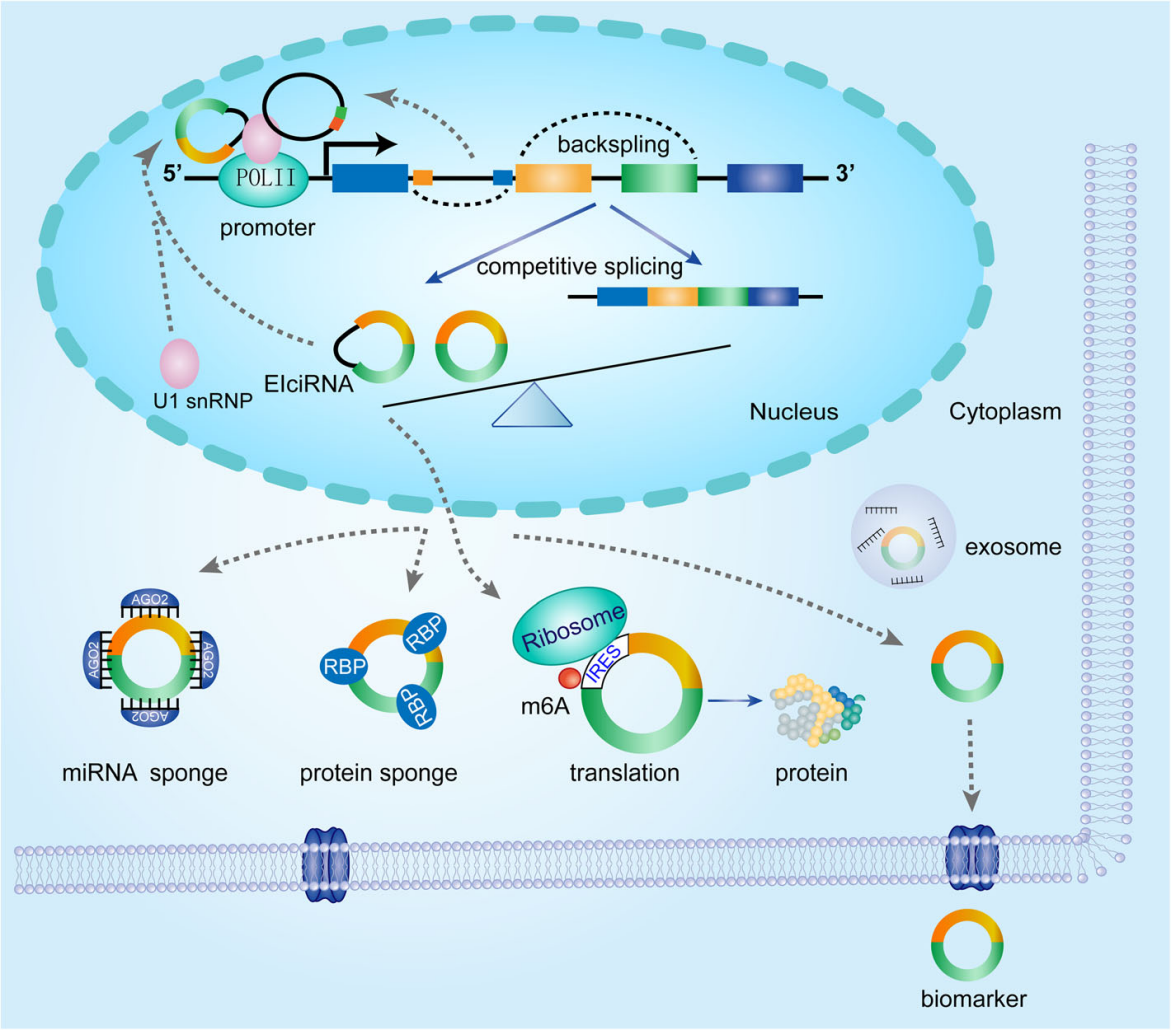
Potential functions of circRNAs. CircRNA can act as miRNA sponges and subsequently regulate relevant target gene expression. CircRNA can regulate transcription and splicing of their parental genes. CircRNA can bind to several proteins to mediate their actions. Abundant IRES and m6A modification can promote multiple circRNAs to translation, especially abundance in GBM. CircRNAs also function as molecular biomarkers existing in serum as well as other body fluid to highlight the significant values for diagnosis and treatment of disease, including cancer

MiRNA-mediated dysregulation of mRNA and relevant signaling pathways are closely associated with progression and treatment resistance for cancer. CircRNAs can function as competing endogenous RNA (ceRNAs) or miRNA sponges to inhibit miRNA and therefore up-regulate the expression of target genes by MREs [48] (Fig. 2). The vast majority of back-splicing circRNAs are predominantly localized in the cytoplasm [25], suggesting the potential as a ceRNA. The best well-known circRNA ciRS-7(circRNA sponge for miR-7), derived from the vertebrate cerebellar degeneration related 1 (CDR1) antisense transcript, was highly expressed in brains and contained more than 70 conserved miR-7 target sites [36]. Overexpression of ciRS7 firmly bound miR-7 to facilitate the specific miR-7/AGO2 interaction, thus attenuated the availability of miR7 to bind to its target mRNAs. Importantly, the function of ciRS7 was conserved, knockdown of miR-7, or upregulation of ciRS-7 impaired midbrain development in zebrafish [24, 36]. The testis-specific circRNA, sex-determining region Y (circ-Sry), dependent on intron pairing-driven circularization, contained 16 miR-138 binding sites and acted as miR-138 sponge in mouse testis [6, 36]. Also, circRNA HIPK3 exhibited sponging multiple miRNAs, including tumor suppressor miR-124, miR-7, miR-4288, miR-654 [46, 49–51]. Previous studies indicated that circ_0034642 and circ_0076248 promoted glioma proliferation and invasion in the miR-1205/BATF3 axis and miR-181a/SIRT1 axis, respectively [52, 53]. It seemed that these findings support the notion that the roles of circRNAs as miRNA sponge may be a general phenomenon. However, there was controversial that miR-671 and miR-7 form a regulatory network via sponge function in the brain, and the combination of ciRS-7 and miR-671 triggers AGO2-mediated cleavage of ciRS-7 rather than miR-7 inhibition [54, 55]. Besides, most circRNAs had fewer binding sites for miRNAs, indicating that multiple circRNAs might not function as miRNA sponges. Therefore, more detailed studies are necessary to clarify the effect of circRNA interaction on inhibitory or scaffold role for miRNAs.

### Nuclear circRNAs regulate transcription and splicing

CiRNA and EIciRNAs mainly produced from processed intron lariats (ciRNAs) and back-splicing with retained introns respectively, and they are dominantly located in the nucleus and regulate gene expression at the transcriptional and post-transcriptional level [28, 29], however, how this type of circRNA is exported or accumulated to the nucleus remain poorly understood.

Ci-ankrd52 and ci-SIRT7 were able to interact with elongating pol II complex and positively modulate the transcriptional activity of their parental genes [28]. EIciRNAs could interact with U1 small nuclear ribonucleoproteins (snRNP) and RNA PoII in the promoter region of the parental gene to enhance gene expression in cis. For instance, blockage of circEIF3J and circPAIP2 reduced the transcriptional level of host genes [29]. In brief, these studies suggest the crucial role of Pol II transcription regulation by nuclear circRNAs, although the relevant mechanisms of action remain unclear.

The processing of circRNAs naturally affected alternative splicing of their linear cognates and competed with pre-mRNA splicing, suggesting that the negative relationship between circRNA and their linear isoforms [34, 56]. For example, circMbl derived from the circularization of the second exon of the MBL, and it can compete with linear MBL mRNA to maintain the balance between canonical splicing and circRNAs production [34]. However, in addition to exon skipping, whether there are additional regulators that affect exon circularization remains to be explored. Another nuclear-retained circSEP3 also was identified to regulate splicing of its linear counterpart through RNA:DNA hybrid or R-loop [57], these studies abovementioned demonstrated that some localized nuclear circRNAs could modulate gene transcription and splicing (Fig. 2).

### CircRNAs interact with RBPs

CircRNAs could bind to the different proteins to form specific circRNA-protein complexes (circRNPs) that subsequently regulates the action of associated protein, the subcellular localization of proteins as well as the transcription of parental or related genes (Fig. 2). The study from Schneider provided biochemical evidence for circRNPs of distinct sizes existed in mammalian cells [58]. Specifically, circ-Foxo3 could interact with senescence-related proteins ID1 and E2F1, and stress elated proteins HIF1a and FAK, facilitating the locations of these transcriptional factors in the cytoplasm and accordingly increased stress-induced senescence and apoptosis in human cancer cells [59]. Another study demonstrated that ectopic expression of circFoxo3 repressed cell cycle progression by binding to the cell cycle proteins cyclin-dependent kinase 2 (CDK2) and cyclin-dependent kinase inhibitor 1 (p21), leading to the formation of a circRNAfoxo3-p21-CDK2 ternary complex [60]. Another example still stemmed from the study of circ-Foxo3 by Du et al. They showed that circ-Foxo3 was minimally expressed in cancer cells and tumor samples and circ-Foxo3 promoted MDM2-induced p53 ubiquitination degradation in the form of circFoxo3-Mdm2-p53 complex to avoid MDM2-induced Foxo3 ubiquitination with low binding affinity, resulting in increased levels of Foxo3 protein [61]. Additionally, circRNAs can also medicate translation of its cognate mRNA via specifically binding to translational factors. For example, circPABPN1 prevented HuR binding to PABPN1 mRNA and hence inhibited its translation by competitively binding to HuR, which was necessary for PABPN1 expression [62]. These data indicated that specific circRNA could combine with different RBPs, and certain circRNAs also have a dynamic affinity to different RBPs. However, how the excellent combination and function between circRNA and RBPs needed to further clarify.

Furthermore, the most recent report from Barbagallo proposed that circSMARCA5, predicted to be enriched in the splicing factor Serine and Arginine Rich Splicing Factor 1 (SRSF1) protein-binding sites, acted as sponge for SRSF1 in GBM [63], raising some questions that whether this type of sponge function of RBPs is similar to circRNA-miRNA interaction and what characteristics should circRNAs have to bind to specific types of proteins or miRNAs. Undoubtedly, some studies suggest that circRNAs have fewer RBP binding sites than coding regions and 3’UTR of their linear mRNAs [19], also indicating that this high affinity for RBPs is not accurately the same as a miRNA sponge function.

### CircRNAs can be translated

The majority of circRNAs produced from middle exons of pre-mRNA and mainly located in the cytoplasm, revealing a lack of evidence of encoding proteins [27]. But, increasing evidence suggests that circRNAs had tremendous potential in a cap-independent manner. Indeed, engineering circRNA with an IRES can be translated in eukaryotic cells [22, 64]. Ribosome footprinting reads from fly heads demonstrated that specific circRNAs, generally sharing the start codon with the hosting RNA, were related to translating ribosomes, and circMbl embedded with IRES was able to encode a protein which was modulated by starvation and FOXO [65]. circRNADb, a comprehensive database for human circular RNAs with protein-coding annotations, showed that 16,328 circRNAs were annotated to have putative ORF longer than 100 amino acids, 7170 of which had IRES elements, 46 circRNAs from 37 genes were verified to have their corresponding proteins [66]. In addition to abundant IRES and ORF responsible for circRNA translation, Yang et al. confirmed that N6-methyladenosine (m6A) s was also enriched in circRNAs and could promote efficient initiation of protein translation. Moreover, translation efficiency was enhanced by methyltransferase METTL3/14 and repressed by demethylase FTO [67]. Besides, this paper also uncovered several short sequences could also drive the effective translation of circRNAs in endogenous text, consistent with Pamudurti [65], revealing circRNA translation could be driven by other mechanisms, such as the methylation of adenosine [67]. Accordingly, the factors involved in circRNA translation process are necessary to further identify.

Cap-independent translation through IRESs was derived and enhanced in cancers, which was subjected to diverse stress such as hypoxia, nutrient deprivation, and genotoxic stress [68]. Besides, many observations from Pamudurti proved that circRNAs translation by cap-independent manner might be meaningful in the brain, partly due to the general start codon between translational circRNA and the hosting RNA as well as location in synaptosome [65]. These findings promote us to speculate that circRNAs translation may be more prevalent in human cancer cells, including gliomas. Furthermore, circRNA and peptides/proteins encoded by these circRNAs played an important in progression and invasion of tumor cells. However, it was stunning to find that the studies of peptides encoded by circRNAs mainly were innately associated with glioma tumorigenesis, indicating the great significance of circRNA in glioma malignant progression. Zhang et al. proposed that circ-SHPRH, used overlapping genetic codes to generate a ‘UGA’ stop codon, resulting in the translation of the 17 kDa SHPRH-146aa. Both circ-SHPRH and SHPRH-146aa were highly expressed in normal human brains and are down-regulated in glioblastoma. SHPRH-146aa protected full-length SHPRH from degradation by the ubiquitin-proteasome, leading to inhibition of cell proliferation [69]. Another typical example was circ-FBXW7, which was generated by back-splicing from exons 3 and 4 from the FBXW7 gene, encoded a novel 21-kDa protein termed FBXW7-185aa. Upregulation of FBXW7-185aa inhibited proliferation and cell cycle acceleration via decreasing the half-life of c-Myc through antagonizing USP28-induced c-Myc stabilization. Besides, circ-FBXW7 and FBXW7-185aa were down-regulated in glioblastoma samples and circ-FBXW7 expression positively associated with glioblastoma patient overall survival [70]. In addition to these reports, recently, Zhang et al. confirmed that circPINTexon2, generated from long intergenic non-protein-coding RNA p53-induced transcript (LINC-PINT), could encode a PINT87aa with 87-amino-acid peptide. PINT87aa directly interacted with polymerase associated factor complex (PAF1c) and suppressed the transcriptional elongation of multiple oncogenes such as CPEB1, SOX-2, c-Myc [31].

Bagchi et al. demonstrated that the protein from circ_12152, derived from human chromosome chr9: 87482157e87570432, participated in glioblastoma progression [71]. Moreover, AKT3-174aa, encoded by circ-AKT3, competitively interacted with phosphorylated PDK1, inhibited GBM progression [72]. Combined with the above-mentioned reports, peptides/proteins encoded by circRNAs usually contain less than 100 amino acids and play critical roles in regulating tumor energy metabolism, EMT transition, the stability of the c-Myc oncoprotein. Consequently, these peptides/proteins represent promising drug targets for tumor treatment or biomarkers for predicting patient clinical prognosis, especially in GBM [16] (Fig. 2).

### Other functions

S. Tan et al. discovered that circRNA F-circEA, produced from the EML4-ALK fusion gene, acting as a novel liquid biopsy biomarker for non-small cell lung cancer [73]. Dong et al. proposed that stable circRNAs can be retro-transcribed and ultimately inserted back into the host genome as processed pseudogenes, had the potential to reshape genome architecture by providing additional CTCF-binding sites. However, the molecular mechanism of circRNA retro-transposition deserved further elucidated [74], Moreover, Holdt et al. demonstrated that circANRIL, binding to peccadillo homologue 1 (PES1), impaired exonuclease-mediated pre-rRNA processing and ribosome biogenesis [75].

### CircRNA and glioma

CircRNAs are more enriched in neuronal tissues and exhibit differential expression in different brain areas. This phenomenon might be attributed to the fact the abundance of protein-coding genes producing various circRNAs and splicing factors and RBPs regulating circRNA formation [18]. CircRNAs are involved in several nervous system diseases such as Alzheimer’s disease mediated by ciRS-7 sponging miR-7 [76]. In addition, growing studies highlight the function of circRNAs on glioma progression and tumorigenicity.

### Expression of circRNA in gliomas

Mounting evidence revealed that aberrant expression of circRNAs implicated in the development of glioma. By RNA sequence, Xu et al. screened for circRNAs with differential expression between three glioma tissue samples and three paired normal tissue samples in combination with five methods, CIRCexplorer2, circRNA-finder, CIRI, find-circ and MapSplice2, and finally identified 12 common circRNAs [77]. Zhang et al. identified 666 differentially expressed circRNAs in U251 from ribosome nascent-chain complex-bound RNA sequencing (RNC-seq) with a false discovery rate (FDR) of ≤0.01 and a fold-change ≥2 [31]. Moreover, Zhu et al. detected the expression profiles of circRNAs in five GBM in an attempt to identify potential core genes in the pathogenesis. The results also confirmed that a total of 1411 differentially expressed circRNAs were identified in GBM patients, including 206 up-regulated circRNAs and 1205 down-regulated circRNAs, suggesting dysregulation of circRNAs was nearly related with the biological process [78]. By high-throughput sequencing data for three paired glioma tissue and normal brain tissue samples downloaded from the Gene Expression Omnibus (GEO) database, Li et al. proved that 493 and 254 circRNAs were identified by FindCirc and CIRC_FINDER, respectively [78]. Using the similar method and the same specimen number, Yuan et al. found 2038 circRNAs were often altered between glioblastoma and matched normal brain tissue. Among these differentially expressed circRNAs, 2002 circRNAs were down-regulated, and 36 circRNAs were up-regulated. Additionally, the construction of co-expression networks suggested that altered circRNAs, enriched in multiple cancer-related pathways, acted as drivers of brain cancers or neurological diseases [79]. The newest study from N. Vo compiled a human cancer circRNA landscape using exome capture transcriptome sequencing, including glioma, providing a valuable resource for the development of circRNAs as diagnostic or therapeutic targets across cancer types [10]. Ruan et al. identified circRNA specificity through detecting across ~ 1000 human cancer cell lines from CCLE polyA-enriched RNA-seq data, including glioma cells [47]. Various specific circRNAs have been identified and analyzed based on vindication experiments, suggesting abnormal expression of circRNAs had potential therapeutic value.

### CircRNAs act as microRNA sponges on the proliferation and invasion

**Fig. 3:**
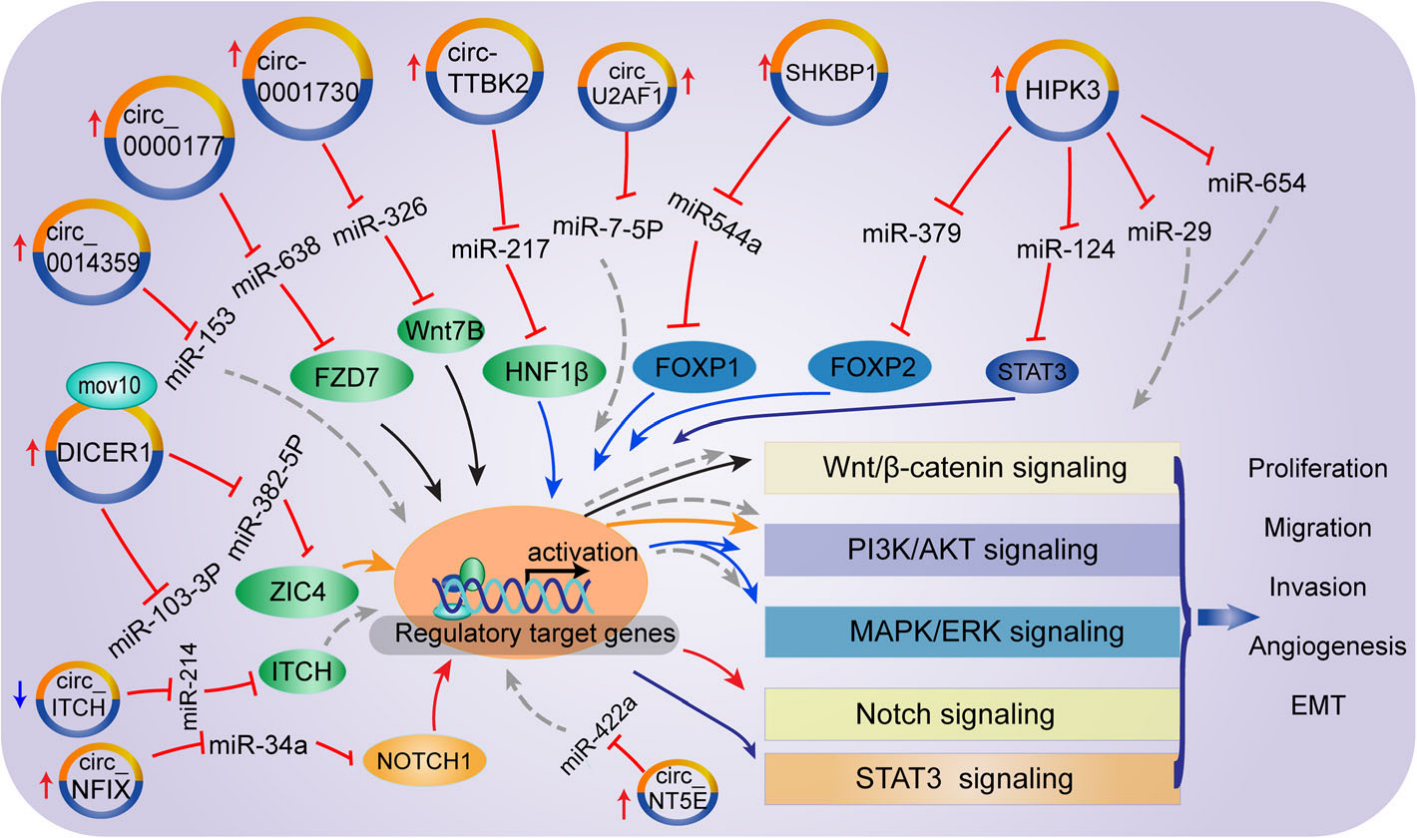
The representative diagram of circRNA mediated ceRNA network and oncogene signaling pathways. The current study in glioma highlights the regulatory relationship of the circRNA-miRNA-mRNA network for different signaling pathways. Multiple identified circRNAs function as miRNA sponge and subsequently up-regulate relevant target genes expression level. However, these target genes or proteins further regulate downstream factors associated with cancer signaling pathways via acting as transcriptional factors or regulatory proteins and other mechanisms. The drawing mainly shows that circ-0000177 activates the wnt/β-catenin pathway through miR-638/FZD7. circTTBK2, circSHKBP1 and circHIPK3 activate PI3K/AKT and MARK/ERK signaling pathway through miR217/HNF1β, miR544a/FOXP1, and miR379/FOXP2 respectively. Additionally, circNPIX activates the Notch signaling pathway through miR34a/NOTCH1. More importantly, a large number of miRNAs, including miR-124, miR-29, and miR-654 sponged by circHIPK3, affect several signaling pathways, suggesting circRNA mediated ceRNA network plays crucial roles in glioma progression through signaling pathways

CircRNA-miRNA-mRNA network not only broadened the understanding of molecular mechanisms but also exhibited significant influence in malignant phenotype, including proliferation, invasion, apoptosis and metastasis. For example, circRNAHIPK3 could sponge multiple miRNAs, containing miR-654 and miR-124-3p [46]. Jin et al. proved that miR-654 was identified as a target of circHIPK3 while miR-654 targeted IGF2BP3, circHIPK3 promoted glioma progression through the circHIPK3/miR-654/IGF2BP3 network [50]. Another study demonstrated that overexpression of circ-HIPK3 promoted proliferative and invasive capacities of glioma cells by sponging miR-124-3p up-regulating STAT3 level [80]. Wang et al. identified circRNAMMP9, also sponging miR-124, was up-regulated in GBM and promoted malignant progression through the miR124/CDK4/AUPKA axis. Meanwhile, they found that eukaryotic initiation factor 4A3 (Eif4A3), which binds to the MMP9 mRNA transcript, induced circMMP9 cyclization in GBM [81]. CircNT5E consists of 7 exons (exons 3–9) from the NE5E genome, affected apoptosis, invasion, and migration abilities of glioblastoma cells via sponging miR-422a, subsequently up-regulating the NT5E, SOX4, PI3KCA, p-Akt, and p-Smad2 levels. Furthermore, they also revealed that ADARB2 bound the pre-mRNA of NT5E and rescued the ADAR1-induced down-regulation of circNT5E, uncovering upstream regulatory mechanism [82]. The study of Li et al. indicated that circ_0046701 was significantly up-regulated in glioma tissues and cell lines and its knockdown remarkably repressed cell proliferation and invasion through upregulating miR-142-3p, resulting in downregulation of ITGB8 [83]. MiR-671-5p, encoded by a gene localized at 7q36.1, was proved to be a region amplified in GBM. Li et al. confirmed that circ_0001946 inhibited the expression of miR-671-5p, thus up-regulating the expression of CDR1, suggesting that activation of the circ_0001946/miR-671-5p/ CDR1 axis may be a potential therapeutic target for GBM treatment [84]. Also, Barbagallo et al. identified CDR1-AS, CDR1, VSNL1 as downstream miR-671-5p targets via a combined in silico and in vitro approach, however, how the CDR1AS activate the transcription of CDR1 need to further explored [85]. circ-0012129 also was reported to significantly increased in glioma tissues, and its knockdown significantly suppressed the proliferation and invasion abilities of U373 and SHG44 gliomas lines through upregulating the expression of miR-661 [86]. circCPA4, circSCAF11, circ-PITX1 were significantly up-regulated in GBM, accelerated the glioma tumorigenesis via let7/CPA4, miR421/SP1/VEGFA, miR-379-5p/MAP3K2 axis, respectively [87–89], while CircMTO1 markedly down-regulated and inhibited GBM proliferation through miR92/WWOX pathway [90]. Additionally, circPTN promoted glioma growth and stemness through sponging miR-145-5p and miR-330-5p, subsequently increased Nestin, CD133, SOX9, and SOX2 expression [91]. In short, circRNA-miRNA-mRNA interaction networks might play vital r*o*les in glioma development and progression (Fig. 3).

### CircRNAs affect the proliferation and invasion of gliomas through cancer-associated signaling pathways

Multiple signaling pathways were innately connected with the initiation and progression of gliomas. Aberrant expression of circRNAs played vital roles in proliferation, cell cycle, invasion and metastasis of glioma through modulating complementary miRNAs or target mRNAs that were firmly related to cancer-associated signaling pathways, such as PI3K/AK/mTOR, Wnt/β-catenin, Notch pathways. Yang et al. verified that cZNF292 silencing repressed glioma proliferation and cell cycle progression via the Wnt/β-catenin pathway, moreover, they also proved that the transcription factors E2F1, NF-κB, HIF-1, AP-1, STAT3 and STAT5 expression markedly down-regulated after cZNF292 silencing, suggesting that the potential mechanism of NF-κB and STAT3/5 pathways by cZNF292 [92]. Similarly, Chen et al. demonstrated that circ_0000177 was up-regulated in glioma cell lines, and its overexpression promoted glioma proliferation and invasion through FZD7-induced activation of Wnt pathway by sponging miR-638 [93]. Circ 0001730 activated the Wnt/β-catenin pathway via the miR-326/Wnt7B axis [94]. PI3K/AKT and ERK pathways are directly related to malignant biological behaviors and treatment resistance. The study from Zheng revealed that circ-TTBK2 promoted proliferation and inhibited apoptosis acting as miR-217 sponge in a sequence-specific manner. Moreover, HNF1β was a direct target of miR-217 and activated Derlin-1 via binding to its promoter via PI3K/AKT and ERK signaling pathways, suggesting blockage of circ-TTBK2/miR-217/HNF1β/Derlin-1 axis may be a potential therapeutic target for human gliomas [95–97]. Furthermore, circ-0014359 and circNT5E promoted glioma progression via PI3K/AKT/mTOR signaling pathway by miR-153 and miR422a, respectively [82, 98]. MiR-7-5p exerted a tumor-suppressive function in glioblastoma via regulation of the EGFR, PI3K/AKT, Raf/MEK/ERK, and IGF-1R pathways [99, 100]. Li et al. indicated that circ-U2AF1 promoted glioma malignancy via inhibiting NOVA2 by sponging has-miR-7-5p [101]. Meanwhile, Circ-CFH was significantly upregulated in glioma tissue and was positively correlated with tumor grade, and it markedly increased the proliferative ability of glioma cells by specifically sponging miR-149 and releasing AKT1 [102]. Besides, dysregulation of the notch signaling pathway mediated by circRNA participated in progression of glioma. For instance, Xu et al. investigated that circNFIX enhanced glioma progression through the upregulation of target gene NOTCH1 via the Notch signaling pathway by sponging miR-34a-5p [77]. Collectively, cancer-related pathways are critically associated with tumorigenesis, chemotherapy and radiation resistance. We believe that more circRNAs identification involved in cancer pathways is valuable to further understand the molecular biology of glioma and develop novel targeted treatments (Fig. 3).

### CircRNA and angiogenesis

It is well acknowledged that hypoxia and various signaling pathways stimulate tumor angiogenesis. Moreover, hypoxia could induce circRNA production, which was also involved in angiogenesis [103]. Repression of cZNF292 suppressed glioma tube formation via the Wnt/β-catenin pathway and related genes such as EGFR, VEGF-A, and the VEGF-A receptor VEGFR-1/2 in human glioma U87MG and U251 cells [92]. FOXP1/FOXP2 promoted angiogenic factor with G patch and FHA domains 1 (AGGF1) expression at the transcriptional level, which promotes angiogenesis via PI3K/AKT and ERK1/2 pathway [104]. He et al. described that circ-SHKBP1 was up-regulated in glioma microvessels and GECs, and negatively regulated the expression of FOXP1/FOXP2 by targeting miR-544a/miR-379. Hence, circ-SHKBP1 modulated glioma angiogenesis through targeting miR-544a/FOXP1/AGGF1 and miR-379/FOXP2/AGGF1 pathway [105].

RBPs can interact with circRNAs to participate in the regulation of the angiogenesis of tumors. For instance, FUS, belonging to the FET(FUS/EWS/TAF15) protein family, could regulate the expression of 19 circRNAs via binding to introns flanking the splicing [106]. Specifically, FUS binds to circ_002136 through RNA-IP and RNA pull-down assays, and inhibition of FUS or circ_002136 dramatically suppressed tube formation of U87 glioma-exposed endothelial cells (GECs). Moreover, knockdown of circ_002136 reduced SOX13 expression by sponging miR-138-5p, whereas SOX13 bound to the SPON2 promoter region to promote expression of SPON2, which promoted angiogenesis. More importantly, SOX13 could enhance FUS transcription via promoter region, uncovering angiogenesis regulation mediated by the feedback loop of FUS/circ_002136/miR-138-5p/SOX13 in glioma [107]. Besides, another report indicated that MOV10 binding circ-DICER1 regulated angiogenesis of glioma via negative regulation of miR-103a-3p/miR-382-5p on ZIC4 in GECs. ZIC4 could lead to downstream target Hsp90β upregulation which promoted tube formation of GECs by activating PI3K/Akt pathway [108]. A recent study proposed that circSMARCA5 acts as sponge for the splicing factor Serine and Arginine Rich Splicing Factor 1 (SRSF1) that targeted and mediated VEGFA expression in GBM, subsequently exhibiting anti-angiogenic function [63] (Fig. 3).

### CircRNAs are potential novel biomarkers for diagnosis and prognosis of gliomas

The identification and functional researches of circRNAs suggest that circRNAs serve as oncogenes or tumor-suppressors governing downstream target genes and have significant clinical applications. In addition to unique covalently circular structure compared with other ncRNAs, there are several other unique characteristics. Specifically, 1) the structural character without 5’caps and 3’poly(A) tails makes circRNAs resistant to exonuclease, and the half-life is longer than 48 h [109] 2) The sequences of most circRNAs are evolutionarily conserved across different species [65], 3) circRNAs show the cell-type, tissue-type and developmental-specific expression [110], 4) circRNAs are produced by exclusive variable splicing and abundant in the cytoplasm of eukaryotic cells [36], 5) circRNAs show diversity and plays regulatory roles at the transcriptional or post-transcriptional level [24], 6) most circRNAs are non-coding, but a few can be translated into polypeptides [111], 7) circRNAs are abundant and widely enriched in exosomes [112]. From clinical perspectives, circRNAs are confirmed to widely exist in blood and fluids (saliva, urine, synovial fluid). Therefore, all these properties promote circRNAs to act as valuable biomarkers for diagnosis, prognosis as well as therapeutic evaluation of glioma.

To date, the association between circRNA expression in tissues or peptides/proteins encoding by circRNA and clinical parameters have been largely reported. Circ_0034642, circ_0074362, cir-ITCH, circHIPK3 and circCPA4 linked to clinical severity and poor prognosis in patients with glioma [50, 53, 87, 113, 114]. Wang et al. proposed that the reduction of circ_0001649 was linked to larger tumor size and advanced WHO grade, indicating circ_0001649 may be an independent prognostic marker after surgery. Moreover, up-regulated circ_0001649 facilitated apoptosis by regulating Bcl-2/caspase-3 pathway [115]. Current researches have shown that peptides/proteins encoded by non-coding RNA represented promising biomarkers for predicting the prognosis of cancer patients [16]. SHPRH-146aa, encoded by circRNA SHPRH, was abundantly enriched in normal human brains and reduced in GBM. Further, it reduces tumorigenicity through protecting full-length SHPRH, which ubiquitinates proliferating cell nuclear antigen (PCNA) as an E3 ligase. AFBXW7-185aa encoded by circ-FBXW7 repressed glioma destructive behaviors via antagonizing USP28-induced c-Myc stabilization. Both of them are negatively related to short survival time of patients [69, 70]. The study from Zhu revealed that the upregulation of circBRAF was an independent predictive factor with good progression-free survival and overall survival in glioma patients by Cox analysis [78].

Extracellular vesicles (EVs, exosomes, and microvesicles) play critical roles in intercellular communication by transporting molecules into the surroundings, thereby altering or reprogramming tumor microenvironment. They contain carriers of various types of molecules such as proteins/peptides, mRNAs, non-coding RNAs and DNA, which determine their roles in tumor progression, metabolic regulation, immune modulation, angiogenesis, therapy resistance [116]. What is more, concerning their high stability and specific differential expression patterns, they are used as promising biomarkers in cancer [117]. For instance, miR-21 and miR-221 were regarded to be highly abundant in CSF-derived EVs and serum-derived exosomes of glioblastoma patients, respectively [118, 119]. Furthermore, Oliver D et al. also highlighted the fact that exosomes had significant clinical implications in central nervous system tumors, especially GBM [120]. However, circular RNAs are extensively enriched and stable in exosomes [121], although the study and application of Exo-circRNA in gliomas are still in its infancy, suggesting serum/CSF Exo-circRNA may act as potential biomarkers, therapeutic resistance as well as delivery of targeted drug molecules. ZHAO et al. identified 63 significantly upregulated and 48 downregulated circRNAs in RR EVs compared with those from U251 cells (Nor EVs). Combined with the prediction of the circRNA-miRNA network, they also proposed that circATP8B4 from RR EVs could be transferred to typical U251 cells and acted as a miR-766 sponge to promote radioresistance [122] (Table 1).

**Table 1:** Representative circRNAs and related signaling pathways in glioma

circRNA	Dysregulation	Sponge target/mechanism	Downstream genes and signaling pathway	Phenotype	Clinical significance	References
circPINTexon2	down	Encode PINT 87aa	Work as an anchor of PAF1 complex and inhibit downstream genes CPEB1,SOX2,c-myc	Tumorigenicity	WHO grade	[31]
circHIPK3	up	miR-654	IGF2BP3	Proliferation, invasion	Prognostic biomarker	[50]
hsa_circ_0076248	up	miR-181a	SIRT1	Tumorigenesis Apoptosis, invasion	TMZ sensitivity	[52]
circ_0034642	up	miR-1205	BATF3	Proliferation, migration, invasion, apoptosis	Prognostic predictor	[53]
circSMARCA5	down	SRSF1	VEGFA	Migration, angiogenesis	WHO grade Prognostic biomarker	[63]
circSHPRH	down	Encode SHPRH-146aa	Protect SHPRH which ubiquitinates PCNA	Proliferation, tumorigenicity	Prognostic biomarker	[69]
circFBXW7	down	Endode FBXW7-185aa	Reduced the half-life of c-Myc by antagonizing USP28-induced c-Myc stabilization	Proliferation, cell cycle	Prognostic biomarker	[70]
circNFIX	up	miR-34a-5p	Notch1 Notch signaling	Proliferation, migration, Invasion, apoptosis	/	[77]
circHIPK3	up	miR-124-3p	STAT3	Proliferation, invasion,	Prognostic biomarker	[80]
circMMP9	up	miR-124	CDK4, AUPKA	Proliferation, migration, Invasion	/	[81]
CircNT5E	up	miR-422a	NT5E,SOX4,PI3K,P-AKT, p-smad2 PI3K/AKT signaling Smad2 signaling	Proliferation, migration, invasion	/	[82]
hsa_circ_0046701	up	miR-142-3P	ITGB8	Proliferation, invasion	/	[83]
has_circ_001946	down	miR-671-5p	CDR1	Proliferation, migration, Invasion, apoptosis	/	[84]
hsa_circ_0012129	up	miR-661	/	Proliferation, migration, invasion	/	[86]
cZNF292	up	/	Wnt/β-catenin signaling and related genes including cyclinA,p-CDK2,VEGFR, EGFR	Proliferation, cell cycle angiogenesis	/	[92]
hsa_circ_0000177	up	miR-638	FZD7 Wnt/β-catenin signaling	Proliferation, migration, invasion	Prognosis biomarker	[93]
circTTBK2	up	miR-217	HNF1β/Derlin1 PI3K/AKT and ERK signaling	Proliferation, migration, Invasion, apoptosis	/	[97]
hsa_circ_14359	up	miR-153	p-AKT PI3K/AKT signaling	Proliferation, migration, Invasion, apoptosis	/	[98]
circU2AF1	up	miR-7-5P	NOVA2 PI3K/AKT and ERK signaling	Proliferation, migration, Invasion, apoptosis	WHO grade	[101]
circCFH	up	miR-149	AKT1 PI3K/AKT signaling	Proliferation	WHO grade	[102]
circSHKBP1	up	miR-544a/miR379	FOXP1/FOXP2/AGG1 PI3K/AKT and ERK signaling	Proliferation, migration, angiogenesis	/	[105]
has_circ_002136	up	miR-138-5p	SOX13/SPON2	Migration, invasion angiogenesis	/	[107]
circDICER1	up	miR-103a-3p miR-382-5p	ZIC4/HSP90 PI3k/AKT signaling	Proliferation, migration, angiogenesis	/	[108]
has_circ_0074362	up	miR-1236-3P	HOXB7	Proliferation, migration, invasion	/	[113]
cir-ITCH	down	miR-214	ITCH/wnt/β-catenin signaling	Proliferation, migration, invasion	Prognostic biomarker	[114]
hsa_circ_0001649	down	/	BCL2/caspase3 signaling	Apoptosis	Tumor size WHO grade Overall survival	[115]

Currently, miRNA medicated TMZ sensitivity through regulating specific target genes have been widely verified. For example, miR-181b regulated the chemosensitivity of glioma to temozolomide by targeting Bcl-2 and EGFR [123, 124]. Also, exosomal miR-1238 may confer chemoresistance in the tumor microenvironment, indicating that circulating miR-1238 acted as a promising therapeutic target for TMZ resistance in GBM [125]. Lei et al. demonstrated that down-regulated hsa_circ_0076248 could remarkably promote the TMZ chemotherapy sensitivity via sponge miR-181a, which could suppress the expression of silent information regulator 1 (SIRT1) [52]. All these researches tend to make circRNAs become promising diagnostic/prognostic biomarkers and novel therapeutic targets.

### CircRNA and immunotherapy in gliomas

PD-1/PD-L1 checkpoint blockade represents a promising target strategy for several types of tumours, including glioma. The report from Chen indicated that PD-L1 modulated immune cell infiltration in glioma microenvironment (TME) and served as signaling protein control multiple pathways including STAT3, PI3K/AKT/mTOR, Ras/ERKsignaling pathways [126]. However, based on low immunogenic responses and immunosuppressive microenvironment of glioblastoma, targeting PD-1/PD-L1 checkpoint is inefficiency mainly attributing to the tumor TME, such as various genomic subtypes or molecular profiles although upregulated PD-L1 is a prognostic biomarker of immune therapy on glioblastoma cells [126, 127]. Therefore, combinational checkpoint blockade immunotherapy with RT, TMZ, antibodies is urgently needed. The relationship between miRNAs and tumor immunity is explicit, while circRNA is closely associated with antitumor immunity based on the theory of binding to miRNAs, proteins, Besides, circRNAs in tumor exosomes serve as tumor antigens to activate antitumor immunity, indicating a potential target for immunotherapy. For instance, miR-138 could target CTLA-4 and PD-1 to escape from immune checkpoint therapy in glioma [128], hence,circ_002136 may also has a potential immunotherapy target through sponging miR138 [107], IL-6R by circHIPK3-miR124 axis may affect gliomas immune response [80], circ_0076248 could participate in immune response by regulating expression of p53 and SIRT1 mediated by miR-181a [52], Furthermore, miR-34a overexpression remarkably weakened PD-L1-induced chemoresistance, supporting that miR-34a is a negative regulator of PD-L1 signaling in glioma [129]. Based on the fact miR-34a could be sponged by circNFIX, suggesting circNFIX may play essential roles in immunotherapy of gliomas [77]. These diverse theories are predictive and the regulatory mechanisms of circRNA-miRNA-mRNA and other available RBPs on glioma immunity regulation are still weak and worthy of comprehensive and in-depth exploration.

### Research strategies and identification

High-throughput RNA-seq and microarray analysis facilitate circRNAs detection. The bioinformatics community rapidly developed the process circRNA exploration that is from custom scripts working on mapped reads to the production of complete workflows [130]. The current protocol, CircleSeq allows computational tools to detect even lowly expressed circRNAs. Besides, a new approach termed RPAD, combined poly-A-depletion and RNase R digestion with polyadenylation, contains an optional rRNA removal step. Indeed, there are multiple tools which address circRNAs detection and function analysis. Some tools, such as find-circ, CIRI, MapSplice, CIRCexplorer, circRNA finder, and Acfs, UROBORUS [131], most of these algorithms largely dependent on unique back-splice junctions (BSJs), which may determine the reliability of circRNA identification based on the complete overlap between circRNA sequences and their cognate linear RNAs. However, these tools not only have various prediction outcomes but also have relatively low effectiveness of filtering step, so combination or renewal of these tools will be more useful to examine and annotate circRNAs. Recently, CIRI2, an updated version of CIRI, integrates advanced matching and detection algorithms to optimally explore the primary data source for circRNAs. Accompanied by rapid development process, more available approaches are presented based on mature read alignment software, which are classified into three main categories including indirect, multi-stage approaches, approach directly employing chimeric reads as well as Tools using statistical approaches. These approaches provide insights into the actual internal structure and first possible functional aspects [130]. Besides, a new report from Zheng presented the CIRI-full software, a novel approach for effective reconstruction of full-length circRNAs based on reverse overlap (RO) feature-based method. In addition to determination of their internal structures similar to the abovementioned, this approach could facilitate genome-wide full-length circRNA identification which is helpful to downstream analyses and identify low-abundance circRNAs more efficient and accurate compared with BSJ feature-based method. Moreover, this isoform-level quantification highly reflects circular isoforms changes between normal and tumor samples. Of course, each approach currently has its limitations. The RO-based method need longer reads to acquire an entire circRNA sequence [132].

Experimental validation is fundamental to further study circRNAs function following computational prediction. qRT-PCR based on divergent primers, which flanking the BSJ sites, was employed to confirm that circular form was amplified in complementary DNA (cDNA) but not genomic DNA (gDNA) whereas sanger sequencing identified the junction sites. Following that, RNase R is a 3′ to 5′ exoribonuclease that degrades nearly all linear RNA but does not digest circular RNA and is used to further verify circRNAs [133]. Finally, northern blotting is a more stringent way based on the specific probe [134]. There are also some limitations, some linear RNAs with the same sequences as those through BSJ sites may be theoretically amplified by qRT-PCR, suggesting other existing mechanisms such as trans-splicing and template switching [135, 136]. Otherwise, some studies demonstrated that some abundant linear RNAs were still detected while some circRNA also were digested after prolonged RNase R treatment [26, 109, 136]. Currently, a combination of these experiments may be the most valuable strategy. However, since existing computational methods were not able to altogether contain the whole circle sequence and complexity of internal structure reconstruction, we speculate that these approaches may be more advantageous for relatively shorter circRNAs(~ 500 bp), needing more elaborate biological validation experiments [130], more accurate or improved methods with higher sensitivity and precision are further explore to address relevant questions.

Knockdown and overexpression of genes are similarly applied to research of circRNAs function. SiRNA or shRNA complementary to the cognate linear RNA through back-splicing sites have potential influence on expression of linear RNA, demonstrating that strict control siRNA half-sequence (∼10 nt) replacement should be carried out [137]. Targeting intronic ICS by the CRISPR/Cas9 system could be designed to disrupt circRNA expresssion [138]. In parallel, circRNA overexpression could be accomplished via in trans from a plasmid construct and in cis from genome-editing tools [137]. Moreover, RNA fluorescence in situ hybridization (FISH) was performed to determine circRNAs subcellular localization for further analysis and bioinformatics analysis, a luciferase reporter assay, RIP, RNA pull-down as well as mass spectrometry are applied to analyze circRNA-miRNA and circRNA-protein interactions [139].

### Dababase of circRNA

In order to facilitate the analysis of circRNA research, several databases have been developed to provide tremendous valuable information. Circ2Traits is categorized based on disease-related SNPs, AGO interaction site and relative miRNAs to highlight potential relationships between circRNAs, miRNAs, and diseases [140]. CircBase is a comprehensive database containing published circRNA expression from distinct samples of several species, also identifying novel circRNA from RNA-seq data [141]. CircNet provides information associated with tissue-specific expression, sequence features, and circRNA-miRNA- mRNA regulatory network [142]. CircInteractome provides bioinformatic analysis of binding sites on circRNA and miRNA, RBP also is helpful to the design of primers and siRNA for specific circRNA [143]. StarBaseV2 (ENCORI) presents a global view on interactions of the RNA-RNA and RNA-protein interactions [144]. CIRCpedia v2, is an updated database for comprehensive circRNA annotation from over 180 RNA-seq datasets across six different species [145]. CircRNADb provides detailed information of the circRNA, including genomic information, exon splicing, genome sequence, internal ribosome entry site (IRES), open reading frame (ORF) and references [66]. CSCD provides prediction of the microRNA response element sites and RNA binding protein sites for each circRNA, predicting potential open reading frame for translatable circRNAs [146]. ExoRBase is a repository of circRNA, long non-coding RNA (lncRNA), and messenger RNA (mRNA) derived from RNA-seq data analyses of human blood exosomes [147]. Recently, N. Vo et al. presented a new cancer-related database containing circRNA from human cancer cell lines as well as tumor samples that is a valuable resource for the development of circRNAs as diagnostic or therapeutic targets across cancer types [10]. CircAtlas, including 44 normal tissues of three species with an average of 72.6% being successfully assembled into full-length transcripts for each species, provided abundant resources with circRNA and its host genes expression values and miRNA and RBP prediction information [148] (Table 2).

**Table 2:** Online databases

Name	Website	Description	References
Circ2Traits	http://gyanxet-beta.com/circdb/	A disease-associated circRNA database providing putative interaction networks miRNA-mRNA	[140]
circBase	http://circbase.org/	A comprehensive unified database of circRNA expression with potential identifying novel circRNAs	[141]
CircNet	http://syslab5.nchu.edu.tw/CircNet/	A database generating an tissue-specific expression and integrated network between circRNA, miRNA and gene	[142]
circInteractome	https://circinteractome.nia.nih.gov/	A web tool exploring miRNA and RBP binding sites on specific circRNA	[143]
StarBasev2 (ENCORI)	http://starbase.sysu.edu.cn/starbase3/	A database identifying RNA-RNA and protein-RNA interactions	[144]
CIRCpedia v2	http://www.picb.ac.cn/rnomics/circpedia	A database used to browse, and download alternative back-splicing events with expression characteristics in various cell types/tissues, including disease samples	[145]
circRNADb	http://reprod.njmu.edu.cn/circrnadb.	A comprehensive database for human circular RNAs with protein-coding annotations.	[64]
CSCD	http://gb.whu.edu.cn/CSCD	a database for the first comprehensive cancer-specific circRNA	[146]
exoRBase	http://www.exoRBase.org	A web-accessible database providing the annotation, expression level and possible original tissues in human blood exosomes	[147]
MiOncoCirc	https://mioncocirc.github.io/.	MiOncoCirc provides a reference of the circular RNA landscape across 40 cancer types.	[10]
CircAtlas	http://circatlas.biols.ac.cn/	A study of circRNA’s variable splicing, conservativeness and relationship with linear RNA	[148]

### Perspective

With the prompt advance of deep-sequence and operational formulas, the mystery of circRNA is gradually uncovered. It is generally recognized that circRNAs currently play vital roles in various physiological and pathophysiological processes linked to the central nervous system. CircRNAs are more enriched in brain and often derived from genes for neuronal and synaptic function whereas less abundance in gliomas, it seems that identifying upregulated circRNAs may have more important clinical value, which is consistent with present researches. However, the regulatory mechanisms of circRNAs biogenesis are complicated. We suppose that high-confidence detection with low false-positive rate and high specificity through improving algorithms may exhibit profound significance into interpreting specific function and circRNA regulatory network in progression of glioma. Besides, it is stunning that circRNAs with translational potential and clinical relationships are prominently found in GBM, to a large extent, due to widely circRNA formation with IRSE by MBL or other regulatory proteins such as enriched splicing factors that are highly enriched in brain, although the precise and specific mechanism is unclear. More critical, peptide and protein encoded by circRNAs provide a considerable number of opportunities for GBM treatment responses, prognosis evaluation, and small-molecule peptide drug developments based on high specificity and activity, low immunogenicity and less cytotoxicity. Moreover, nanoparticle-based strategies are sufficient to help the delivery of specific compounds to brain tumors across the blood-brain barrier. Therefore, targeted circRNAs and relevant peptides combined with nanoparticle-based cerebral drug-delivery systems will represent a significant new perspective for further treatment of glioma.

Currently, specific expression and functional studies have advocated that circRNAs are strictly related to the WHO grade and prognosis of gliomas patients, suggesting its potential diagnostic value. However, these studies dominantly focus on minimal clinical-pathological samples; there are no relevant reports from body fluids, especially blood and CSF. The particular traits containing more stability, long half-life as well as abundance in exosomes tend to deduce that Exo-circRNA from blood and CSF of GBM patients provides excellent insights into non-invasively diagnosis and distinguish different gliomas subtypes, as well as early treatment assessment with high sensitivity combined with magnetic resonance imaging or other traditional biomarkers. Therefore, the expression profile analysis and relevant roles research from body fluids are urgent to further explore molecular pathology of glioma medicated by circRNA.

Epigenetics is involved in numerous critical natural processes and plays significant roles in the occurrence and progression of cancers. A variety of circRNAs have been identified to modulate the epigenetic alterations, such as DNA methylation and histone modifications. For instance, circFECR1 could recruit TET1 DNA demethylase to the FLI1 promoter and subsequently led to CpG DNA demethylation [149]. However, in turn, epigenetics can also affect circRNAs biological formation. Ferreira et al. demonstrated that cancer-specific 5′-end CpG island hypermethylation could silence linear and circular RNAs expression and confirmed that aberrant DNA methylation profiles of circular and linear RNA loci are common events in tumorigenesis [150], indicating the expression of circRNAs could be epigenetically regulated. These studies highlight a circle regulatory network comprised of DNA epigenetic alterations/cicRNAs /mRNA(DNA methylation enzymes). In the last several years, epigenetic modifications of RNA, including m6A, 5-methylcytosine (m^5^C), pseudouridine, m7G, N1-methyladenosine, attracted increasing attention. Considerable evidence verified that RNA epigenetic modifications play a vital role in CNS diseases, including glioma. It is demonstrated that m6A modification induced highly cell-specific expression, and m6A circRNAs have long single exons [151]. Park et al. revealed that m6A recognized the binding of the protein YTHDF2 to the target molecule and recruited HRSP12 to mediate the cleavage of circRNA by the RNA endonuclease RNase P/MRP complex, suggesting critical significances to perceive circRNAs dynamic change [152]. Also, a new study from Chen et al. demonstrated that the nuclear export of circNSUN2 is mediated by YTHDC1 in an m6A methylation-dependent manner [153]. All data highlights the significance of epigenetic alterations regulating the biogenesis and metabolism of circRNAs. Besides, it has been reported that UAP56 and URH49 were identified to modulate the export of long and short circRNAs in HeLa cells [154, 155]. Meanwhile, a methyl-guanosine cap and poly(A) tail also were studied to determine RNA exportation from the nucleus [156]. Despite AGO2/miR-671-mediated cleavage of CiRS-7 has been confirmed and extracellular vesicles or microvesicle release may also a type of cleavage of circRNA in mammalian cells [157]. However, there are no definite answers about how are circRNAs transported within the cells and the mechanisms of ultimate degradation. Whether or how other RNA modifications such as m5c participate in the biological regulation of circRNAs remain unknown. Otherwise, there are other several outstanding issues about the specific mechanisms about circRNA competing with linear splicing of p re-mRNA, and cis- or trans-regulators and relevant regulatory mechanisms affecting circRNA biogenesis under distinctive biological settings. The answers of all these questions will contribute to a new interpretation of circRNAs biology and undoubtedly enhance the comprehensive understanding of their roles.

The previous revealing mechanisms implicated in the tumorigenesis of gliomas majorly were paid more attention to the miRNA sponge, protein/peptides encoding associated with signaling pathways, EMT transformation. Undoubtedly, multiple circRNAs are located in the nuclear; accordingly, other potential functions, including regulating gene transcription, protein interactions or sponges, remain to be deeply addressed. Based on high heterogeneity of gliomas, circRNAs with specificity under discrete molecular subtypes are imperative to further discovered. Additionally, more precise methods changing circRNA expression levels but not affecting their residing genes also remain to be clarified.

## Conclusions

In conclusion, there is accumulating evidence regarding the crucial role of circRNAs in the initiation and progression of glioma, but circRNAs research is still in its infancy. We believe, accompanied by the development of research strategies and sequencing technologies, further elucidation for circRNAs implicated in the tumorigenesis of gliomas will eventually accelerate the clinical application of circRNAs in the diagnosis, treatment, prognosis evaluation.

## Data Availability

Not applicable.

## Abbreviations

c-myc: Transcriptional regulator Myc-like
CPEB1: Cytoplasmic polyadenylation element binding protein 1
CTLA-4: Cytotoxic T-lymphocyte associated protein 4
DHX9: DExH-box helicase 9
E2F1: E2F transcription factor 1
EMT: Epithelial mesenchymal transition
FOXO: FOXO transcription factor
FUS: FUS RNA binding protein
GBM: Glioblastoma multiforme
IGF-1R: Insulin like growth factor 1 receptor
IGF2BP3: Insulin like growth factor 2 mRNA binding protein 3
IL-6R: Interleukin 6 receptor
IRES: Internal ribosomal entry site
ITGB8: Integrin subunit beta 8
lncRNA: Long non-coding RNA
miRNA: microRNA
MOV10: Mov10 RISC complex RNA helicase
MREs: miRNA response elements
ORF: Open reading frame
PCR: Reverse-complementary repeats
RBPs: RNA binding proteins
SOX13: SRY-box transcription factor 13
SOX2: SRY-box transcription factor 2
SPON2: Spondin 2
STAT3: Signal transducer and activator of transcription 3
VSNL1: Visinin like 1
YY1: YY1 transcription factor
ZIC4: Zic family member 4
